# C-Peptide Versus Insulin: Relationships with Risk Biomarkers of Cardiovascular Disease in Metabolic Syndrome in Young Arab Females

**DOI:** 10.1155/2012/420792

**Published:** 2012-08-01

**Authors:** A. Abdullah, H. Hasan, V. Raigangar, W. Bani-Issa

**Affiliations:** College of Health Sciences, University of Sharjah, P.O. Box 27272, Sharjah, UAE

## Abstract

*Background*. Obesity is a major health concern and is associated with metabolic syndrome (MetS) that increases the risk for cardiovascular disease (CVD). Since little is known about the relationships between MetS components and CVD in overweight/obese young Arab females, our study aimed at examining these relationships and further to explore the associations between connecting peptide (C-peptide) and insulin with these biomarkers. *Subjects and Methods*. In this cross-sectional study, 80 apparently healthy young Arab females were recruited and grouped by their body mass index (BMI) into normal-weight (GI) and overweight/obese (GII) groups. *Results*. The two groups significantly differed in BMI, waist circumference (WC) and values of biomarkers, namely, leptin, fasting insulin, uric acid (UA), insulin resistance (HOMA-IR), C-peptide, high-sensitivity C-reactive protein (*hs*-CRP), high-density lipoprotein cholesterol (HDL-C), systolic blood pressure (SBP), and diastolic blood pressure (DBP). C-peptide significantly correlated with WC, leptin, UA, and HDL-C and was predicted by three biomarkers; UA, WC and HDL-C. Whereas, insulin significantly correlated with only two biomarkers including leptin and DBP and was predicted by UA and DBP. *Conclusions*. The present study highlighted the association between MetS and CVD in young Arab females and the possible role of C-peptide in the prediction of CVD.

## 1. Introduction

In spite of considerable efforts around the world for the prevention and treatment of cardiovascular diseases (CVD), they still remain to be the number one cause of death [1]. This could be attributed to the rapidly increasing incidence of obesity, a well-known risk factor for insulin resistance (IR), diabetes mellitus (DM), atherogenic dyslipidemia, and elevated blood pressure. Obesity is now a major public health concern, especially because it could lead to “metabolic syndrome” (MetS), which is a cluster of risk factors for CVD and type 2 DM of metabolic origin [2].

The International Diabetes Federation (IDF) updated their criteria for MetS in 2006 and defined it as the presence of central obesity as the essential element measured either by race/ethnicity specific waist circumference (WC) thresholds or a body mass index (BMI) >30 kg/m^2^ and any two of the following: raised triglycerides (TG) >150 mg/dL, reduced high-density lipoprotein cholesterol (HDL-C) <50 mg/dL, raised systolic blood pressure (SBP) >130 mm Hg or diastolic blood pressure (DBP) >85 mm Hg, or raised fasting blood glucose (FBG) >100 mg/dL. For research purposes the IDF recommends extra criteria in their “Platinum standard” definition to predict CVD in MetS. These extra criteria include abnormal body fat distribution, atherogenic dyslipidmia (beyond elevated TG and HDL), dysglycemia, IR (other than elevated FBG), vascular dysregulation (beyond elevated blood pressure), proinflammatory state, prothrombotic state and hormonal factors [3].

One of the other names applied to this constellation of findings of MetS is “the IR syndrome.” IR characterized by hyperinsulinemia and hyperglycemia, and change in levels of adipocytokines that could lead to vascular endothelial dysfunction, an abnormal lipid profile, hypertension, and vascular inflammation, all of which promote the development of CVD [4–6]. It is well documented that high levels of insulin are associated with elevated “connecting peptide” (C-peptide) levels as both are produced in equimolar amounts [7, 8]. C-peptide was considered an inert substance, but recent studies have proven it is a bioactive peptide [9] that affects various cell membranes, including endothelial, renal and nerve cells [10]. The physiological effects of C-peptide are different from and complementary to those of insulin [11]. High levels of insulin and C-peptide coexist and are suggested to promote atherogenesis thus contributing to increased risk for CVD [12].

Insulin resistance in obese individuals may also be associated with high levels of plasma leptin and low levels of adiponectin. Both are adipocyte-derived hormones involved in the pathogenesis of atherosclerosis which may place obese subjects at greater risk for CVD [13, 14].

Some additional biomarkers like high-sensitivity C-reactive protein (*hs*-CRP) and uric acid (UA) could be associated with CVD. High-sensitivity CRP together with other proinflammatory adipokines can facilitate atherogenic lesions and induce IR, hence they may predict coronary heart events in apparently healthy subjects [15, 16]. Uric acid has proinflammatory and proliferative effects on the vascular smooth muscle cells and causes dysfunction of endothelial cells; hence, hyperuricemia may be associated with renal and CVD [17].

Few studies have comprehensively studied the relationship of MetS to other biomarkers (leptin, *hs*-CRP, adiponectin, insulin, C-peptide and UA) in overweight/obese young Arab females. Therefore, this study was undertaken to explore and analyze these associations in greater depth. Additionally, considering the fact that high C-peptide levels coexist with hyperinsulinemia [7, 8], researchers would like to examine the role of C-peptide in prediction of the development of CVD in MetS as compared to insulin. Results of the current study may provide some preliminary data about MetS in this ethnic group, which may add value to ethnic specific criteria proposed by IDF. Also, the current study may support the role of C-peptide in the development of CVD.

## 2. Subjects and Methods

### 2.1. Subjects

This study was carried out in the female clinic at the University of Sharjah, UAE. Because sex differences have been reported in plasma leptin, lipid profile, UA, and BMI, we chose to study females only [18, 19]. Eighty female students of the University of Sharjah aged between 18 and 30 years were included in this cross-sectional study. Height, weight, WC, SBP, and DBP were measured. Female participants were classified into two groups based on their BMI: Group I (GI) was categorized as normal-weight subjects with a BMI <25 kg/m^2^ considered as the control group and Group II (GII) as overweight/obese subjects with a BMI ≥25 kg/m^2^ [20]. Females having their menstrual cycle during the study period, or known to have chronic illnesses, or using long-term medications were excluded from the study. All subjects read the information sheet and signed the consent form before participation.

The study was approved by the Ethical Committee at the College of Health Sciences, University of Sharjah.

### 2.2. Biochemical Methods

Blood samples of 10 mL were obtained in the morning (11–12 am) by venipuncture after overnight fasting (minimum 12 hours fasting) to estimate the following biomarkers: (1) serum leptin and adiponectin concentrations {enzyme-linked immunoassay (ELISA; SPI-BIO Co., France)}, (2) Insulin and C-peptide (electrochemiluminescence analyzer), (3) FBG (enzymatic reference method with hexokinase), (4) UA, total cholesterol, and TG {enzymatic colorimetric method}, (5) low-density lipoprotein cholesterol (LDL-C) and HDL-C (homogeneous enzymatic colorimetric method), and (6) *hs*-CRP {Immunoturbidimetry}. Testing materials for biomarkers 2 through 6 were from Roche Diagnostics, Germany. 

All samples were processed and examined by the Al-Tiqani Medical Analysis laboratory, which is certified by the “Radox International Quality Assessment Scheme (RIQAS)” using good principles of laboratory practice.

### 2.3. Indexes

The BMI was calculated as body weight (kg)/height (m)^2^. The homeostasis model assessment index HOMA-IR = (fasting plasma insulin (mU/L) × fasting plasma glucose (mmol/L)/22.5) for insulin resistance was calculated [21].

### 2.4. Statistics

Statistical analysis was performed using IBM Statistical Package for Social Sciences (SPSS, version 19). Means and standard deviations (SD) were calculated for all parameters. The independent sample *t*-test was used to compare the means of different variables in the two groups. In addition, the Pearson correlation coefficient (*r*) was used for correlation analysis in the overweight/obese group. Linear regressions were carried out considering insulin and C-peptide as independent variables with other CVD risk factors which correlated significantly with either insulin or C-peptide in the overweight/obese group. A *P* value <0.05 was considered significant.

## 3. Results

Baseline characteristics of participants are summarized in Table 1. Of the 80 subjects (mean age = 21 years, SD = 2.4) studied, a greater proportion (*n* = 45, 56.3%) was overweight/obese classified by BMI (mean BMI = 30.8 kg/m^2^, SD = 4.5) with a mean WC of 83.7 cm (SD = 10.3). This group was labeled as GII, while the remaining were normal-weight (*n* = 35, 43.7%) with a BMI of 21.4 kg/m^2^ (SD = 2.5) and a mean WC of 66.9 cm (SD = 5.2) and labeled as GI. The differences in BMI and WC of the two groups were highly significant (*P* < 0.001).

Similarly, highly significant increase in the values of leptin (46.2%), fasting insulin (38%), uric acid (18.9%), and HOMA-IR (39.8%) with *P* values of <0.001 was noted in GII. Significant differences were also found in levels of C-peptide (14.7%), *hs*-CRP (51.4%), HDL-C (−12.4%), SBP (4.5%), and DBP (6.8%) in GII, (*P* < 0.05). On the other hand, insignificant differences (*P* > 0.05) were found in the levels of FBG, total cholesterol, TG, LDL, adiponectin and, HbA1c between the two groups.

Table 2 shows correlations of C-peptide and insulin with other cardiovascular risk factors in overweight/obese subjects. In GII, C-peptide showed significant positive correlation with WC (*r* = 0.36, *P* = 0.02), leptin (*r* = 0.31, *P* = 0.03), uric acid (*r* = 0.35, *P* = 0.01) and HDL-C (*r* = −0.36, *P* = 0.01). Whereas insulin was found to have two significant positive correlations with leptin (*r* = 0.36, *P* = 0.01) and DBP (*r* = 0.33, *P* = 0.03).

The significantly correlated biomarkers with either C-peptide or insulin or both were included in the stepwise regression analysis. As shown in Table 3, C-peptide is significantly predicted by three factors, namely, UA (*B* value = 1.15, *P* < 0.001), WC (*B* value = 5.28, *P* = 0.02), and HDL-C (*B* = −9.46, *P* = 0.01). With regard to insulin, after adjusting for C-peptide, it was predicted only by UA (*B* value = −0.1, *P* = 0.006) and DBP (*B* value = 0.57, *P* = 0.03).

## 4. Discussion 

Our study focused on examining elements of MetS in normal weight and overweight/obese apparently healthy young Arab females with emphasis on the relationships of insulin and C-peptide with biomarkers of CVD.

Upon comparison of the baseline characteristics of the overweight/obese with the normal weight females in our study, it is apparent that the former group had causative factors implicated in the pathogenesis of MetS; namely central obesity (WC ≥ 80 cm and BMI > 30 kg/m^2^) and increased IR evident by a HOMA-IR value >1.8 *μ*g/mL. As there were no ethnic specific cutoffs for WC of Arab females, the Europid values were considered in this study as suggested by IDF for diagnosis of central obesity [3]. Whereas cutoffs for HOMA-IR were adopted from another study done in a population with similar ethnic background residing in close geographical proximity [22].

Apart from central obesity and IR, considering other recommended factors for the diagnosis of MetS, the overweight/obese group did not demonstrate high TG, low HDL-C, or raised SBP and/or DBP, to the levels that meet the minimum IDF criteria for confirming MetS. This could be attributed to the younger age, healthy nature, and other unknown ethnicity-related factors of our subjects. However, some of these values (HDL-C, SBP, and DBP) were found to be significantly different as compared to our control group. Such findings may place overweight/obese subjects at a higher risk for development of CVD later in life. Considering the paucity of information in the Arab population, particularly in females, it could be estimated that the values specified by the IDF criteria for the diagnosis of MetS may not be fully applicable to this ethnic group.

 Examining the additional metabolic criteria recommended by IDF for clinical research to assist in prediction of CVD, the overweight/obese group showed significantly high leptin, fasting insulin, and *hs*-CRP levels compared to those in the control group. High serum leptin levels may be beneficial for early diagnosis of MetS, as it was found to be correlated with CVD risk and MetS in adults [23]. It is also known that significantly high insulin levels (hyperinsulinemia) with normal FBG are features of IR which may further be implicated in the development of CVD [24]. Also elevated level of *hs*-CRP, evident in our overweight/obese group, is proven to be an inflammatory biomarker and a strong independent predictor of incident CVD [25].

Based on previous literature, it was expected that adiponectin values would be significantly lower in the overweight/obese group [14]. However, these were found to be within the normal range, which could be accounted for by either the small sample size or the genetic makeup of our population; a research with a larger sample size with in depth investigation of the genetics of Arab females should be conducted.

The significantly elevated levels of UA in the overweight/obese group may suggest the importance of UA as an additional marker for CVD, although its value remained within the normal ranges. This could be explained by the fact that the studied population was composed of young females in whom estrogen enhances the excretion of uric acid [26] and keeps it within the normal range.

Study data revealed a higher percentage change in insulin (38%) compared to C-peptide (14.7%) levels in overweight/obese females. Considering that insulin has a shorter plasma half-life as compared to C-peptide [27–29], we would expect to find lower percentage change in insulin rather than C-peptide levels. These findings may be explained by reduced hepatic clearance of insulin in overweight/obese individuals which accounts for higher peripheral insulin levels [28].

Comparing the significant correlations of C-peptide and insulin to other essential/additional criteria for diagnosis of MetS, as recommended by IDF [3], C-peptide exhibited four (HDL-C, leptin, WC, and UA) compared to only two correlations of insulin (DBP and leptin). The numbers and levels of significant correlations of C-peptide could add value to the possibility of its inclusion along with other factors for diagnosis of MetS, which could in turn put overweight/obese individuals at a greater risk for the development of CVD in later life.

The considerable importance of C-peptide was further supported through results of the regression model, which showed that C-peptide can be predicted by CVD biomarkers like HDL-C, WC, and UA; with UA having the highest association whereas insulin can be predicted only by two biomarkers: DBP and UA. This further reemphasizes the role of C-peptide as a possible additional biomarker for CVD in obese subjects [12].

All the above findings signal the warning signs of MetS which doubles the risk of developing CVD and increases 5-fold the risk for type 2 DM [30], further highlighting the possible role of C-peptide as an additional biomarker in the prediction of the early development of CVD in obese subjects in addition to insulin level.

 Considering the seemingly low-risk profile of our studied sample and the burden of obesity combined with DM and CVD in the UAE in recent years, it is imperative that clinicians have an understanding of the components of MetS and reinforce the importance of lifestyle changes at a young age to prevent later development of MetS and CVD. Awareness campaigns are an important step for this region to draw attention to MetS and its impact on health of individuals.

## 5. Conclusion

Our study attempted to add knowledge regarding the development of MetS and CVD in overweight/obese young Arab population. Also, it opens the door for future clinical research to determine additional criteria for diagnosis of MetS and to assist in refining the definition of MetS and to allow comparisons across different ethnic groups.

Furthermore, it draws attention to the considerable role of C-peptide as an additional biomarker in the prediction of the early development of CVD in overweight/obese young Arab females. Importantly, the general population should consider the significance of maintaining a healthy lifestyle particularly at an early age to reduce obesity related disorders later in life.

Finally, this was a relatively small scale study in this ethnic group which could be replicated in a larger sample to provide greater epidemiological evidence for the role of additional biomarkers such as C-peptide in the development of CVD. Considering the multiethnic nature of the UAE residents, comparative studies among different ethnic groups could be conducted to study the effect of lifestyle-related factors on the development of MetS.

## Figures and Tables

**Table 1:** Baseline characteristics of normal-weight (Group I (*n* = 35)) versus overweight/obese females (Group II (*n* = 45)).

Variables	Group I mean (SD)	Group II mean (SD)	Percentage difference (%)	*P* value
Body mass index (kg/m^2^)	21.4 (2.5)	30.8 (4.5)	30.5	<0.001
Waist circumference (cm)	66.9 (5.2)	83.7 (10.3)	20.1	<0.001
C-peptide (ng/mL)	2.1 (0.7)	2.5 (0.7)	14.7	0.02
*hs*-C-reactive protein (mg/L)	2.6 (5.3)	5.3 (5.7)	51.4	0.03
Diastolic blood pressure (mmHg)	68 (9.7)	73 (10.4)	6.8	0.03
Systolic blood pressure (mmHg)	109.8 (9.8)	115 (9.3)	4.5	0.02
Fasting blood glucose (mmol/mL)	4.4 (0.3)	4.5 (0.3)	2.0	0.25
Fasting insulin (*μ*U/mL)	6.5 (3.8)	10.6 (6.0)	38	<0.001
HbA1c (%)	3.9 (0.4)	4.1 (0.4)	4	0.08
Total cholesterol (mg/dL)	150 (26.7)	156 (20.9)	3.9	0.28
Triglyceride (mg/dL)	64.1 (24.8)	73.1 (27.8)	12.3	0.13
HDL-C^∗^ (mg/dL)	63.1 (13.6)	56.1 (10.1)	−12.4	0.01
LDL^∗∗^ (mg/dL)	75.6 (22.2)	83.5 (20.8)	9.4	0.11
Uric acid (mg/dL)	3.3 (0.7)	4.1 (0.9)	18.9	<0.001
Leptin (ng/mL)	20.3 (9.7)	37.7 (14.8)	46.2	<0.001
Adiponectin (*μ*g/mL)	13.4 (1.4)	13.0 (1.1)	−2.5	0.27
HOMA-IR	1.3 (0.8)	2.1 (1.3)	39.8	<0.001

^∗^High-density lipoprotein cholesterol, ^∗∗^low-density lipoprotein cholesterol.

**Table 2:** Coefficients of simple correlation (*r*) of fasting C-peptide versus fasting insulin with studied parameters in overweight/obese subjects (*n* = 45).

	Overweight/obese subjects			
	C-peptide		Insulin	
	*r*	*P*	*r*	*P*
*hs*-CRP	0.06	0.66	0.007	0.96
SBP	0.24	0.10	0.22	0.14
DBP	0.28	0.05	0.33	0.03^∗^
Leptin	0.31	0.03^∗^	0.36	0.01^∗^
HDL-C	−0.36	0.01^∗^	−0.18	0.22
Uric acid	0.35	0.01^∗^	0.07	0.60
WC	0.36	0.02^∗^	0.22	0.14

^∗^Significant correlations (*P* < 0.05).

**Table 3:** Linear regression analyses of fasting C-peptide versus fasting insulin as independent variables and other biomarkers as dependent variables in overweight/obese group (*n* = 45).

	Independent variables			
Dependent variables	C-peptide		Insulin	
	*B*	*P* value	*B*	*P* value
HDL-C	−9.46	0.01^∗^	0.60	0.15
Leptin	0.57	0.91	0.85	0.18
WC	5.28	0.02^∗^	0.38	0.14
DBP	4.2	0.06	0.57	0.03^∗^
Uric acid	1.15	<0.001^∗^	−0.1	0.006^∗^

^∗^Significant (*P* < 0.05).
